# Does the addition of cone-beam CT to panoral imaging reduce inferior dental nerve injuries resulting from third molar surgery? A systematic review

**DOI:** 10.1186/s12903-022-02490-x

**Published:** 2022-11-03

**Authors:** James Robbins, Katelyn Rene Smalley, Pamela Ray, Kamran Ali

**Affiliations:** 1 Specialty Doctor Oral and Maxillofacial Surgery, Royal Cornwall Hospital Treliske, TR1 3LJ Truro, UK; 2 Plymouth University, Drake Circus, PL4 8AA Plymouth, UK; 3 QU Health, College of Dental Medicine, Qatar University, 141 F H-12 Annex Building, 2713 Doha, Qatar

**Keywords:** CBCT, Computed tomography, Third molar, Dental nerve, Nerve injury

## Abstract

**Objective:**

This systematic review aims to examine whether cone-beam CT (CBCT) assessment influences the incidence of nerve injury following high-risk mandibular third molar (MTM) surgery.

**Study Design:**

Randomised controlled trials comparing two and three-dimensional imaging for assessing high-risk MTMs were included. MEDLINE, EMBASE, CENTRAL and the Dentistry and Oral Science Source (DOSS) were systematically searched along with extensive grey literature searches, hand searching of web sites, and detailed citation searching up to 3 September 2022. Risk of bias was assessed against the Cochrane Risk of Bias Tool (RoB 2.0). Certainty of the evidence was assessed using GRADE.

**Results:**

Two authors independently screened 402 abstracts prior to full text screening of 27 articles, which culminated in seven RCTs for inclusion. Two studies were assessed as high risk of bias overall. The other five raised some concerns largely due to unblinded patients and lack of prior trial registration. Just one study reported significantly less nerve injuries following CBCT. The remaining six articles found no significant difference.

**Conclusion:**

The seven RCTs included in this systematic review offered moderate quality evidence that CBCT does *not* routinely translate to reduced incidence of nerve injury in MTM removal. A single study provided low quality evidence for a consequent change in the surgical approach. Low quality evidence from 3 studies suggested CBCT does *not* influence the duration of third molar surgery.

## Introduction

Injury to the inferior dental nerve (IDN) is a known risk of mandibular third molar (MTM) surgery that can be very unpleasant and distressing for patients [1]. Consequently, preoperative radiographic imaging is essential to indicate the anatomical relationship of the tooth with the IDN [2]. Along with proximity to the IDN several panoramic markers have been associated with increased risk of nerve injury, of which three (darkening of the root, diversion of the canal and loss of canal cortication) carry greatest signficance [3]. Where such high-risk radiographic signs are evident, incidence of injury has been quoted as high as 20%, with 4% being permanent [4]. Permanent injuries frequently lead to reduced quality of life and increased depressive symptoms, [5] and up to 70% experience long term pain and disability [6].

Three-dimensional (3D) imaging such as cone-beam computed tomography (CBCT) has an excellent capacity to visualise the anatomical relationship between the tooth and nerve [7]. Understandably therefore, dentists and oral surgeons have increasingly become interested in the ability of CBCT to detail in 3D the anatomical relationship of the IDN to MTM in the hope of reducing iatrogenic injuries during surgery.

An early prospective trial found CBCT to be superior to OPG in predicting vascular bundle exposure after MTM removal with considerable sensitivity (93%) and specificity (77%) [8]. Some favoured that this impressive diagnostic accuracy with CBCT, in combination with an experienced surgeon, could eliminate permanent IDN injury altogether [9]. On the other hand, it has been suggested that OPG is adequate for determining risk of IDN injury [10] and a 6-fold increase in availability of CBCT scanners in Finland did not reduce the incidence of reported nerve injuries over a 5-year period [11].

In recent years the availability of higher quality studies has instigated European Academy of DentoMaxilloFacial Radiology (EADMFR) guidance to be updated [12]. Five randomised controlled trials (RCTs) and a systematic review and meta-analysis provided strong evidence that preoperative CBCT does not reduce postoperative sensory disturbances of the IDN. While this was an important position paper, lack of available funding from the European Commission meant that it appeared to rely upon non-systematic methodology that raises concern for publication bias. This fact, in combination with the time that has now elapsed since its publication, have determined the focus of the present review as a systematic update to this seminal study in answering the following question.

*In adults planned for mandibular third molar surgery, does the addition of cone-beam CT, in comparison to panoral imaging alone, reduce injuries to the inferior dental nerve?*

## Materials and methods

### Inclusion criteria and outcomes

This review was conducted according to the Preferred Reporting Items for Systematic Reviews and Meta-Analyses (PRISMA) guidelines [13]. A stepwise approach, as detailed by Bramer, [14] was adopted to construct a search strategy based on the population, intervention, comparison, outcome and study design (PICOS) concept. Only randomised controlled trials (RCTs) were included to maximise the validity of the conclusions. Included studies had to incorporate patients being planned for removal of one or more high-risk MTMs, as assessed by two-dimensional (2D) radiography (panoral or alternative view). The intervention group of patients had to additionally undergo 3D imaging such as, but not limited to, CBCT. The primary outcome measure was injury to the IDN measured as patient-reported sensory disturbance, or objectively assessed. No restriction was made on the method used to assess injury as there is no consensus method [15]. Secondary outcome measures were permanent nerve injury, defined as signs/ symptoms persisting at six months, change to treatment plan or surgical technique, and duration of surgery. Papers that could not be obtained full text in English language were excluded. Retrospective study designs were excluded especially regarding treatment planning where the surgical decision or technique had to be made by operating clinicians leading to actual treatment.

### Search strategy

The search strategy was constructed for the MEDLINE scientific database and peer reviewed by an information specialist prior to translation for EMBASE, CENTRAL and the Dentistry and Oral Science Source (DOSS). Comprehensive searching of the grey literature was also undertaken including a wide range of trials registries and resources detailing theses, dissertations, and conference proceedings (ClinicalTrials.gov, ISRCTN, OpenGrey, ICTRP, EThOS, DART, OACD, ISI, Zetoc). CBCT manufacturer web sites were hand searched. Finally, the Scopus database was used to manually explore the citations and references of all included studies, as well as those of important preceding papers identified via scoping searches. All searches were conducted on 3 September 2022 and are fully detailed in the supplementary information. No restriction was made upon year of publication.

### Screening and data extraction

All identified papers were imported and deduplicated in the bibliographic management software program EndNote X9 [16]. All screening was undertaken independently by two reviewers (JR and PR) with reference to best practice guidelines [17]. Any disagreements were resolved by discussion. Titles and abstracts were screened against a tested screening tool using Rayaan software before full text screening of included articles. Data extraction tables were then used to manually obtain data from those papers included at full text.

### Quality assessment

Risk of bias for each study and outcome was assessed against the Cochrane Risk of Bias 2 Tool (RoB2) [18]. The quality of the evidence for each outcome was assessed using the Grading of Recommendations, Assessment, Development and Evaluation (GRADE) approach [19].

## Results

**Fig. 1:**
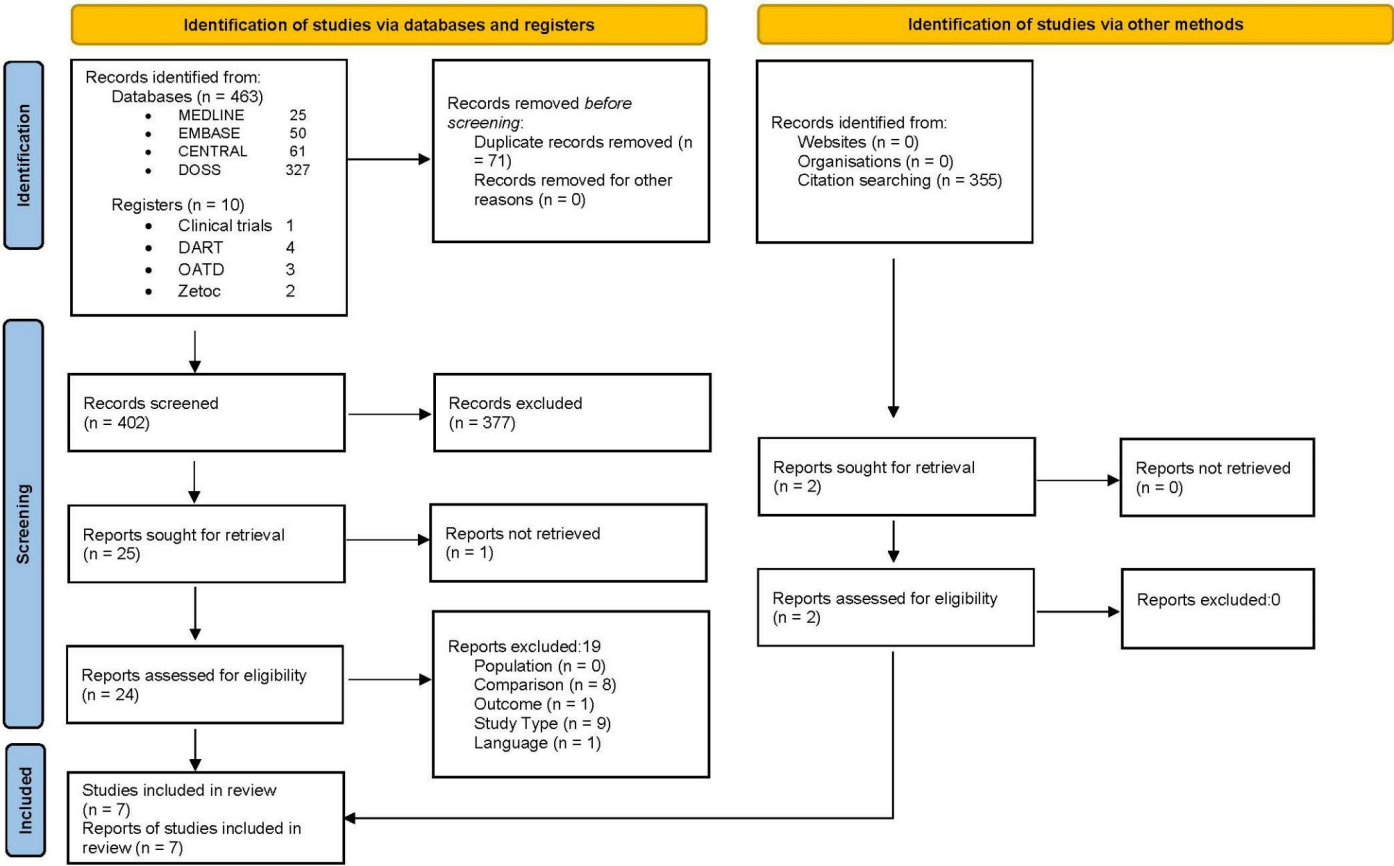
PRISMA flow diagram of the processes leading to the seven articles included for review

Of 402 abstracts identified following deduplication, 27 were agreed for full text screening, which culminated in seven RCTs for inclusion in the review. Two of these were supplementary to those identified in the EADMFR position paper. Details of the 20 omitted papers with reasons for exclusion are provided in Table 1. Figure 1 summarises the processes leading to the inclusion and exclusion of studies via the various searching methods in a PRISMA flow diagram [20].

**Table 1:** Excluded studies and reasons for exclusion

Author & Year	Exclusion code	Explanation
Aravindaksha et al., 2015	ACCESS	Unable to obtain full text
Fee, Wright & Cunningham, 2016	STUDY TYPE	Commentary on Ghaeminia 2015 study [20]
Fusaro et al., 2017	STUDY TYPE	Longitudinal observational cohort study
Gencheva, 2017	STUDY TYPE	Retrospective study
Ghaeminia et al., 2009	COMPARISON	No control group, all received CBCT
Ghaeminia et al., 2011	COMPARISON	No control group, all received CBCT
Jhamb et al., 2009	COMPARISON	No control group, all received CBCT
Jun et al., 2013	STUDY TYPE	Retrospective study
Lenz, 2017	LANGUAGE	Non-English, unlikely relevant
Matzen et al., 2013	COMPARISON	No control group, all received CBCT
Matzen et al., 2019	COMPARISON	No control group, all received CBCT
Matzen et al., 2020	COMPARISON	No control group, all received CBCT
Mendonca et al., 2021	STUDY TYPE	Retrospective study
Neugebauer et al., 2006	STUDY TYPE	Retrospective study
Petersen et al., 2014	OUTCOME	Cost benefit analysis only
Preece, J. 1988	STUDY TYPE	Narrative review
Roeder, Wachtlin & Schulze, 2012	STUDY TYPE	Sample size calculation only
Shiratori et al., 2013	COMPARISON	No control group, all received CBCT
Susarla & Dodson, 2007	STUDY TYPE	Retrospective case series
Tantanapornkul et al., 2007	COMPARISON	No control group, all received CBCT

### Characteristics of included studies

Table 2 presents characteristics of the included studies. All seven included studies adopted a parallel RCT design with patients randomised to either a control group receiving a panoramic radiograph alone to treatment plan their MTM, or an intervention group that underwent a CBCT in addition to a panoral. Populations differed in how authors defined the risk relationship on 2D imaging. All but one study required MTMs to exhibit specific relationships with the IDN on panoramic imaging and the other [21] limited participants to those with horizontally impacted class II, position B MTMs as described by Pell and Gregory [22]. Definition of higher risk relationships varied with little information provided by Mabongo et al. [23] Korkmaz et al. [24] required high-risk signs as described by Rood and Shehab [3] and the remaining studies referenced the degree of overlap of the MTM root and IDN. Petersen, Vaeth and Wenzel [25] stipulated any overlap was sufficient whereas Ghaeminia et al. [26] included only those cases where the root crossed at least half of the depth of the IDN. The two studies by Guerrero et al. [27, 28] required overlap of the root and IDN but excluded cases with high-risk signs such as loss of the radiopaque borders of the canal.

**Table 2:** Included articles for review by location, sample size (number of mandibular third molars) and definition of high-risk relationship with the inferior dental nerve

Authors	Year	Location	Sample size (MTMs)	MTM risk criteria
Guerrero et al.	2012	Leuven, Belgium	86	Radiographic overlap of root over IDC but *excluding* high risk (e.g., loss of white lines of the IDC)
Guerrero et al.	2014	Leuven, Belgium & Lima, Peru	256	As 2012 study above
Ghaeminia et al.	2015	Three centres in the Netherlands	320	Radiographic overlap of root over at least half of IDC height
Badawy et al.	2016	Alexandria, Egypt	20	Horizontally impacted mandibular third molar class II position B*
Peterson et al.	2016	Copenhagen, Denmark	230	Radiographic contact with or overlap of root over IDC
Korkmaz et al.	2017	Trabzon, Turkey	139	One or more radiographic signs described by Rood & Shehab [3]**
Mabongo & Thekiso	2019	Witwatersrand, South Africa	93	“…panoramic signs that suggested close proximity between the tooth roots and the mandibular canal.“

MTM = Mandibular Third Molar, IDC = Inferior Dental Canal. *As described by Pell and Gregory [22]** (1) Loss of the white line of the IDC; (2) darkening of the roots; (3) narrowing of the IDC or roots; (4) dark and bifid roots; (5) deflected roots; (6) diversion of the IDC.

All surgery across the included studies was undertaken under local anaesthetic with careful atraumatic techniques described. Surgeon experience was indicated in all but one paper [21] and three authors analysed experience variability as a risk factor for nerve injury [23, 25, 26]. Five authors specifically described that roots were elevated in a direction that avoided undue pressure on the IDN as guided by the imaging [21, 23, 24, 25, 26].

### Characteristics of outcomes

#### Nerve injury

All seven studies included sensory disturbance as a primary outcome measure assessed seven days post-operatively. Five studies also followed up patients with temporary altered sensation until recovery or, if persistent at six months, diagnosed permanent injury [23, 24, 25, 26, 27].

All seven studies assessed IDN injury using at least the accepted light touch sensation method [29]. In four of the trials, investigators additionally questioned patients regarding subjective change in IDN sensation [21, 23, 24, 26] Only Ghaeminia et al. [26] reported subjective findings independently of objective assessments.

#### Treatment plan

Only one of the studies investigated the effect of imaging method on the treatment plan [24]. Two independent surgeons used the available imaging to agree by consensus whether bone removal or tooth sectioning would be required and in which direction the tooth should be elevated in order to rotate the roots away from the IDN. Guerrero et al. (2014), as a secondary outcome, compared the reliability of the two imaging methods to predict MTM position, root number and apical divergence of the root as assessed by five blinded radiologists.

#### Duration of surgery

Three authors compared the effect of imaging method upon the duration of surgery recorded [24, 26, 27].

The results of all included studies for the primary outcome measure are summarised in Table 3. One study investigated just 20 MTM removals and perhaps not surprisingly, no incidences of nerve injury were seen with either imaging method [21]. Among the remaining six papers, overall incidence of temporary nerve injury ranged from 2.3% ^28^ to 14.8% ^25^ with an overall mean of 7.9%. These figures are broadly consistent with previous studies describing higher risk MTMs [31, 32] and the variation may reflect differences in inclusion criteria for risk factors as detailed in Table 2. For example, Guerrero et al., [28] who observed just 2.3% incidence of nerve injury included ‘moderate risk’ MTMs only, whereas Korkmaz et al., [24] who reported 10.1% incidence, required higher risk features on OPG.

**Table 3:** Included studies by sample size and incidence of nerve injury by intervention group

Author (Year)	Sample size (MTMs)	Overall incidence of nerve injury	OPG		CBCT		P- value
**Guerrero et al. (2012)**	86		43		43		
Cases of nerve injury	2	2.3%	1	2.3%	1	2.3%	1
**Guerrero et al. (2014)**	256		130		126		
Cases of nerve injury	7	2.7%	5	3.8%	2	1.6%	0.45
Permanent nerve injury	No information						
**Ghaeminia et al. (2015)**	320		164		156		
Subjective nerve injury	20	6.3%	9	5.5%	11	7.1%	0.64
Objective nerve injury	16	5%	8	4.9%	8	5.1%	1
Permanent nerve injury	7	2.2%	2	1.3%	5	3.2%	0.27
**Badawy et al. (2016)**	20		10		10		
Cases of nerve injury	0		0		0		1
**Peterson et al. (2016)**	230		116		114		
Cases of nerve injury	34	14.8%	13	11.2%	21	18.4%	0.13
Permanent nerve injury	2	0.90%	1		1		1
**Korkmaz et al. (2017)**	139		67		72		
Cases of nerve injury	14	10.1%	11	16.4%	3	4.2%	0.017*
Permanent nerve injury	0						
**Mabongo & Thekiso (2019)**	93		55		38		
Cases of nerve injury	13	14%	5	9.1%	8	21.1%	0.85
Permanent nerve injury	1	1.1%					

MTM = Mandibular Third Molar, OPG = Orthopantomogram, CBCT = Cone-beam Computed Tomography, * = significant result

Only one author reported a significant difference in nerve injury between intervention and control groups [24]. This paper showed a benefit of additional CBCT imaging with only 4.2% of MTM removals resulting in injury, compared to 16.4% for MTMs planned with OPG only (*P* = 0.017). Perhaps significantly this was the only study that required MTMs to exhibit one of the high-risk radiographic signs [3]. Guerrero et al. [27] also found slightly more nerve injuries in the OPG group, but the difference was not significant (OPG 3.8%, CBCT 1.6%, *P* = 0.45). Three papers recorded higher frequency of nerve injuries in the CBCT group, but the difference was not significant (*P* > 0.05) [23, 25, 26]. The final paper recorded just one injury (2.3%) in each group [28].

#### Permanent nerve injury

Where documented, all studies defined permanent nerve injury as those persisting at six months after surgery. One author provided no information regarding follow up of seven observed temporary injuries [27]. A total of 10 (1.1%) permanent injuries were recorded across the other six investigations incorporating 888 higher risk MTM removals. This was lower than may have been expected from previous studies, particularly for such higher risk MTMs [30, 31]. None of the trials found a significant difference for permanent nerve injury between the two groups.

#### Treatment plan

Only one study compared the effect of CBCT intervention on the surgical plan, ascertaining whether a preferred direction of elevation could be established and also deciding whether the approach would require ostectomy alone or with the addition of crown/ root sectioning, defined by the authors [24] as a complex extraction. In 97.2% of MTMs undergoing CBCT, a preferred direction of elevation was determined to avoid compressing the IDN, which was significantly greater than the 10.4% determinable with OPG alone (*P* = 0.000). Furthermore, significantly more teeth imaged with additional CBCT were planned for complex extraction (*P* = 0.000).

#### Duration of surgery

Three papers investigated the influence of additional CBCT on surgical duration as a secondary outcome measure. Two studies reported mean durations of surgery that were very similar between intervention and control groups and although standard deviations were somewhat narrower in both studies with the addition of 3D imaging, no differences were statistically significant [26, 27] (*P* = 0.91, *P* = 0.65 respectively). Korkmaz et al. [24] on the other hand, curiously categorised their continuous data into either more or less than 20 min operating time, from which they reported that CBCT led to a significant reduction in operating time (*P* = 0.000). No further detail was provided to suggest any clinical significance for this finding however, indicating high risk of measurement and selective reporting bias.

Differences in overall mean duration of surgery in the three studies may be explained by different surgeons and possibly different measurement techniques, which were not detailed by Ghaeminia et al. [26].

### Risk of bias

**Fig. 2:**
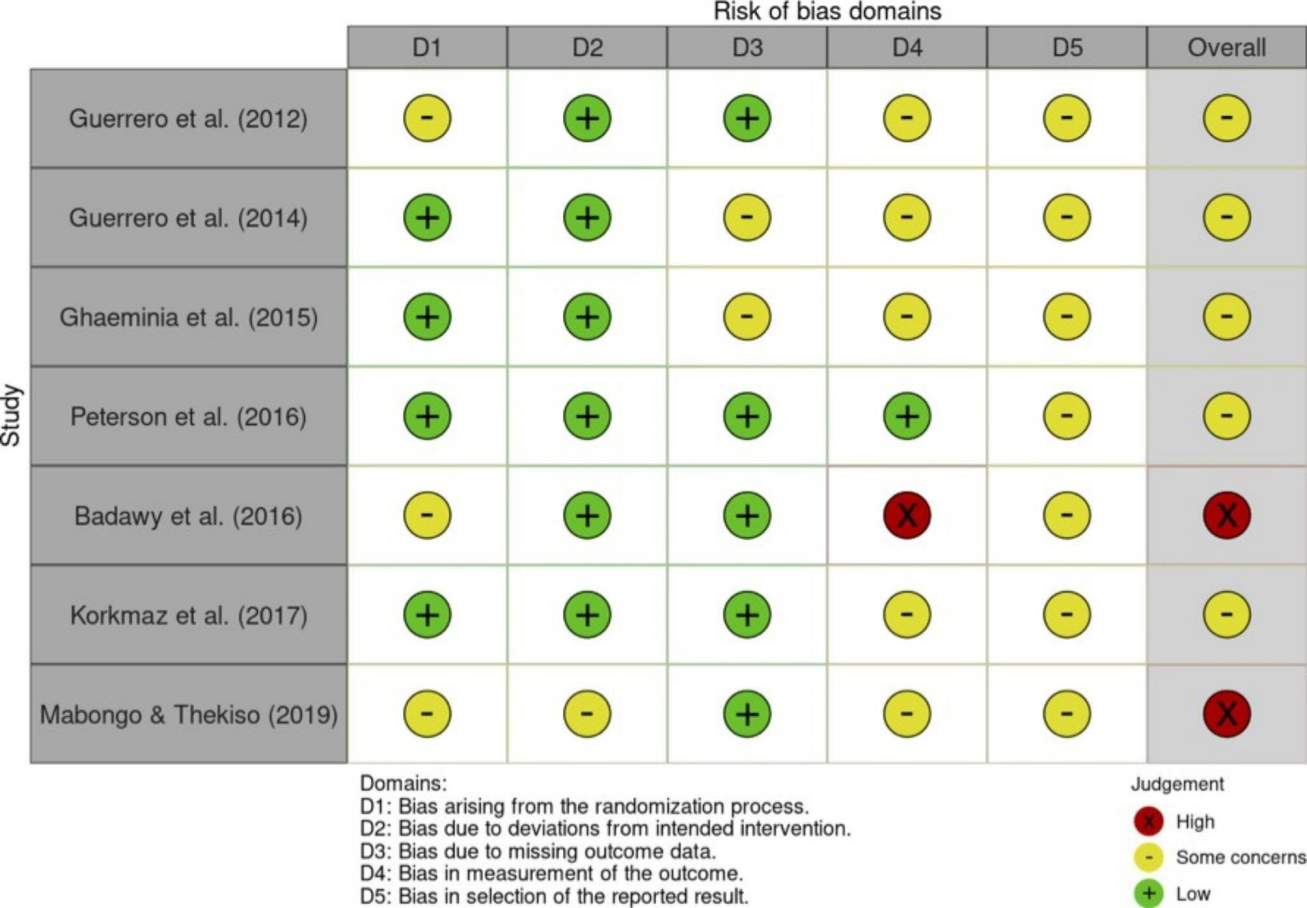
Summary of risk of bias assessment for each study by domain and overall judgement (Primary outcome- Nerve injury). D = Domain.

**Fig. 3:**
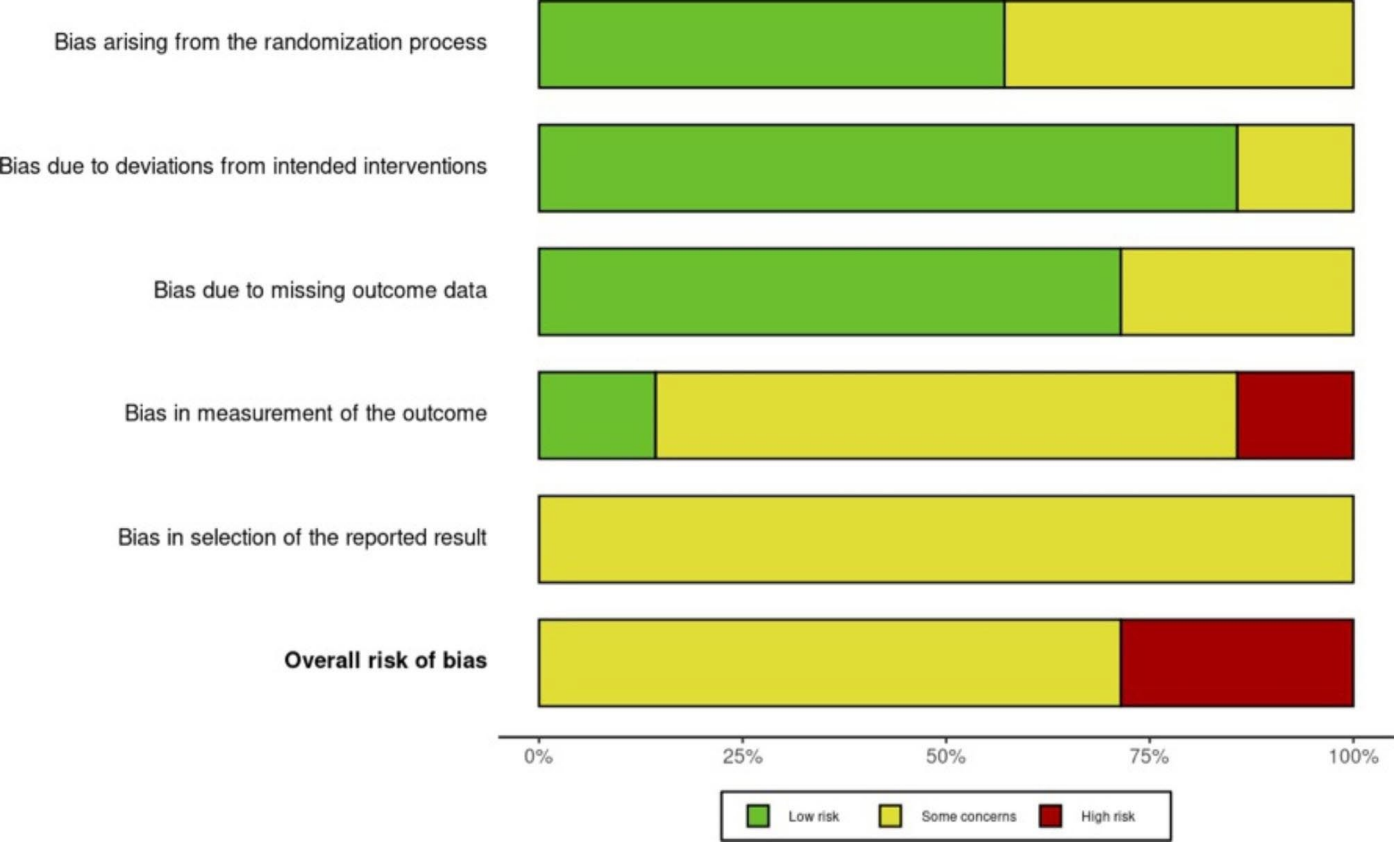
Risk of bias graph for primary outcome nerve injury: assessment of risk of bias in each of the five domains by percentage across all studies

Risk of bias across the seven included studies is summarised in Figs. 2 and 3, which were produced using the ‘robvis’ online tool [32 ]. Figure 2 summarises risk of bias assessments for each study by domain and overall. Two studies were assessed high risk of bias overall however both included small sample sizes that contributed minimally to the overall findings [21, 23]. The remaining five studies raised some concerns in one or more domains that overall did not substantially lower confidence in the results and ‘some concerns’ was concluded overall.

Figure 3 summarises risk of bias assessments by domain. Collectively the included studies raised little concern for bias arising from the randomisation process, from deviations from intended interventions, or from missing outcome data. All studies raised some concerns for reporting bias due to lack of trial registration, and for measurement bias due to unblinded outcome assessors. In all but one study [21] the investigators reviewing patients post-operatively were blinded to the intervention group. Only one study blinded patients to their intervention group via a sham CBCT procedure [25].

#### Treatment plan

Only Korkmaz et al. [24] compared the surgical plan between the two imaging groups, recording the predicted need for ostectomy and tooth sectioning, along with the preferred direction of root elevation to avoid compressing the IDN. Unfortunately, high risk of bias was identified in measurement of the outcome as the two maxillofacial surgeons making the treatment plans were not blinded.

#### Duration of surgery

All three studies comparing surgical time between the two groups were assessed at high risk of bias. Two in measurement of the outcome that was recorded by unblinded surgeons [26, 27] and the other in measurement and selective reporting as results were recorded simply as more or less than 20 min operating time, with no indication of the spread of what should have been continuous data [24].

#### Quality of the evidence (GRADE)

Regarding nerve injury the quality of the evidence was downgraded one level due to study limitations. These centred around unblinded patients acting as outcome assessors and lack of trial protocols to exclude selective outcome reporting. In accordance with GRADE guidelines, this suggests moderate confidence in the effect estimate reflecting the true effect, but with a possibility that it is substantially different [33].

GRADE assessments for both treatment planning and also duration of surgery were downgraded two levels due to very serious concerns regarding study limitations. These largely pertained to unblinded surgeons making treatment plans and recording duration of surgery. This led to an overall judgement of low-quality evidence and limited confidence in the effect estimate, which may be substantially different from the true effect [33].

## Discussion

In summary the findings of this SR demonstrate that even for high-risk MTM cases, the use of CBCT does not appear to routinely correlate with reduced incidence of nerve injury. Weak evidence suggests there is no reduction in surgical duration. Low quality evidence from a single available study indicates that additional 3D imaging may change the surgical approach when planning MTM removal [24].

With regard to nerve injury these findings are consistent with previous SRs [34–36] and an EADMFR position paper [12] indicating that CBCT does not appear to reduce the incidence of IDN injury following MTM removal in most cases. A small field CBCT necessary to image an MTM is associated with approximately a five-fold increase in radiation compared to an OPG [37]. Preoperative costs associated with CBCT are also approximately four times greater [38]. There is consequently general agreement that there is no place for CBCT in the routine radiographic assessment of MTMs, which is also the position of recent RCS guidance [2].

Five of the seven RCTs included in this review risk assessed MTMs based solely on proximity of the root to the IDN [21, 25–28]. The only study to require specific high-risk signs, such as loss of canal cortication, was notably the only study to find a significant reduction in MTM injuries following CBCT assessment [24]. The absence of these high-risk 2D radiographic signs has been correlated with low-risk of nerve injury [10]. Therefore, it is suggested that future RCTs should restrict inclusion criteria to MTMs exhibiting one or more of the highest risk signs (darkening of the root, diversion of the canal, loss of canal cortication) [2, 3].

Given the low incidence of IDN injury it has also been suggested that far larger sample sizes are required to prove superiority of CBCT in assessing MTMs [39]. Unfortunately however, such numbers are unlikely to be practically or financially achievable, and future RCTs may be better focussed upon specific high-risk signs on 2D imaging to try to establish common scenarios where CBCT should be advocated.

Surgical experience is another factor that has been well associated with risk of nerve injury and also the decision upon whether to request 3D imaging [30]. Most extractions in these studies were undertaken by more experienced surgeons, which may be relevant regarding the low overall incidence of nerve injury observed.

### Treatment plan

No RCTs were identified that studied the effect of additional CBCT in guiding a treatment decision in terms of MTM removal, monitoring or alternative strategy such as coronectomy or removal of the opposing tooth. A previous prospective study that asked surgeons to make a treatment decision based first on 2D imaging, and then a CBCT scan, found that treatment decision altered in 12% of cases [40]. Another prospective study found that use of CBCT resulted in a significant proportion of MTMs being downgraded in risk with a consequent change in the surgical approach [41]. Prospective cohort studies such as this may, on reflection, be better suited to investigate the influence of CBCT upon treatment decision and would be a recommended inclusion in future reviews.

Limitations of this review include the restriction to English language papers only, however comprehensive searching of the published and grey literature was undertaken, and evidence suggests that searching for non-English papers rarely changes the conclusion of systematic reviews [42]. Furthermore, a meta-analysis might have been helpful. However, due to the risk of bias in the primary studies, a meta-analysis was not undertaken as it could potentially compound the errors, and result in misleading interpretations.

### So, what is the role of CBCT in MTM management?

The author’s conclusion is that CBCT should be reserved for high-risk cases where the prescriber feels 3D imaging has potential to change the treatment decision, or surgical technique, in a manner that may avoid nerve injury.

To be more objective in this recommendation is difficult. MTMs exhibiting darkening of the root, deviation of the canal or loss of canal cortication on 2D imaging are more likely to be associated with nerve injury [3]. CBCT in these cases may be considered to establish whether there is any canal contact or compression around the root that also correlates with higher risk for injury [43]. Other 2D and 3D signs may also correlate with high-risk for injury and there appears to be no consensus agreement upon an exhaustive list as yet [2]. Where justified and feasible such signs may favour avoiding extraction in favour of monitoring or coronectomy, which may reduce IDN injury by up to 84% [44]. However, treatment planning must also consider the potential immediate and delayed complications of these alternative approaches.

## Conclusion

CBCT should not be used routinely to assess MTMs, and it is unlikely to reduce risk of nerve injury even in most high-risk cases. The decision to undertake CBCT should therefore be carefully justified incorporating individual patients’ expectations and values in identifying whether the added radiation and cost implications are likely to result in a clinically significant change in management.

## Data Availability

Detailed search strategies, data extraction tables, RoB2 and GRADE assessments are available from the corresponding author upon request.
